# Data of cytotoxicity, p53 and Akt downstream proteins and physiological indexes in hepatocellular carcinoma cells or HepG2-bearing nude mouse model administered by α-Humulene

**DOI:** 10.1016/j.dib.2020.105325

**Published:** 2020-02-23

**Authors:** Hao Chen, Ji Hao, Yanzhang Wen, Yibing Lv, Lu Chen, Jingquan Yuan, Xinzhou Yang

**Affiliations:** a Guangxi Scientific Research Center of Traditional Chinese Medicine, Guangxi University of Chinese Medicine, Nanning, 530001, China; b School of Pharmaceutical Sciences, South-Central University for Nationalities, Wuhan, 430074, China; c Guangxi Institute of Medicinal Plant, Nanning, 530023, China

**Keywords:** α-Humulene, Hepatocellular carcinoma, Cytotoxicity, Akt, Side effects

## Abstract

This data article contains data related to the research article entitled “α-Humulene inhibits hepatocellular carcinoma (HCC) cell proliferation and induces apoptosis through the inhibition of Akt signaling” (Chen et al., 2019) [1]. The article focuses on the antiproliferation of α-Humulene (HML) and the mechanisms involved in HCC cells inhibition. In this data, cytotoxicity of HML in several HCC cell lines are reported, together with the changes in proteins involving in p53 and Akt downstream. Weight curve, blood biochemical parameters and organ indices from HepG2-bearing nude mouse model are also provided, suggesting the potential side effects in HML administration.

**Table:** Specifications table

Subject area	Pharmacy
More specific subject area	Anticancer effect of natural products
Type of data	Figures, tables and
How data was acquired	Microplate reader (Thermo Scientific, Vantaa, Finland) at 450 nm. Automatic biochemical analyzer (Beckman Coulter, USA)
Data format	Analyzed and raw
Experimental factors	Huh7, SMMC-7721, HepG2, Hep3B or L-02 cells were exposed to HML (1.25–50 μg/mL), worthmanning or Insulin (100 nM) for 6 or 12 h. HepG2-bearing nude mice models were administered by HML (0–20 mg/kg).
Experimental features	Cytotoxicity of HML to serval HCC cell lines was assessed. The effect of HML on p53 and Akt downstream proteins in HCC cells. Physiological indexes of HepG2-bearing nude mouse model administered by HML.
Data source location	Wuhan, China
Data accessibility	Data is provided in the article.

**Table:** 

**Value of the Data** • Hepatocellular carcinoma (HCC) cells was significantly inhibited by α-Humulene (HML) treatment, which indicated that HML, a natural sesquiterpene, played a cytotoxic role in many essential oils consisting of it showing antineoplastic property on Hepatocellular carcinoma (HCC) cells. • The data evidenced that HML was a potential inhibitor on Akt pathway, which provide a candidate compound in the Akt-related disease. • The data provided the potential side effects represented in the weight loss by HML in mouse model, which cautioned that the application of natural products consisting of HML may cause safety issues. • The blood biochemical parameter or organ index data may allow for comparisons among the HepG2-bearing, non-tumor nude mouse model and other HCC models.

## 1 Data

**Fig. 1:**
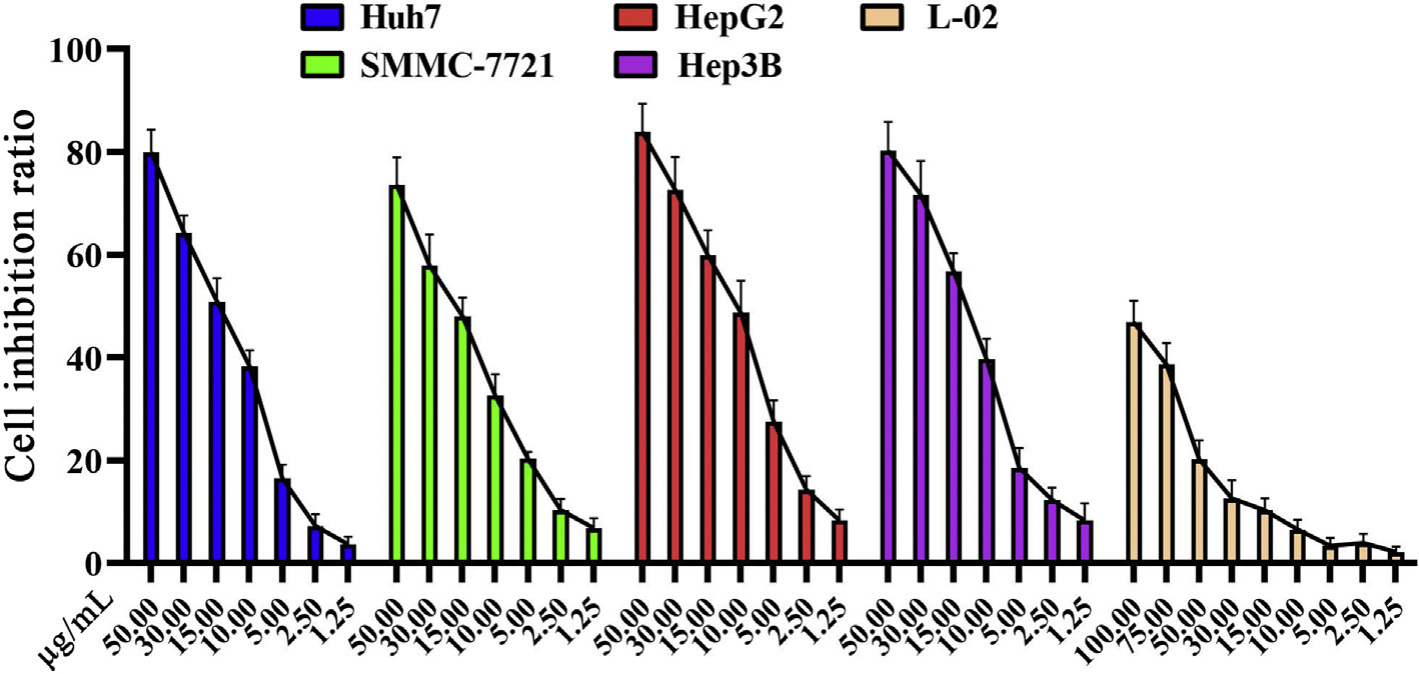
The cancer-selective cytotoxic effects of HML. HCC cells were grown in 96 well plates and treated with HML at 1.25–50 μg/mL for 12 h; Normal hepatocytes (L-02) was treated with HML at 1.25–100 μg/mL for 12 h. Cytotoxicity assessed through CCK-8 assays, and IC_50_ values were calculated. The cytotoxic effects of HML in L-02 were significantly lower in HCC cells.

**Fig. 2:**
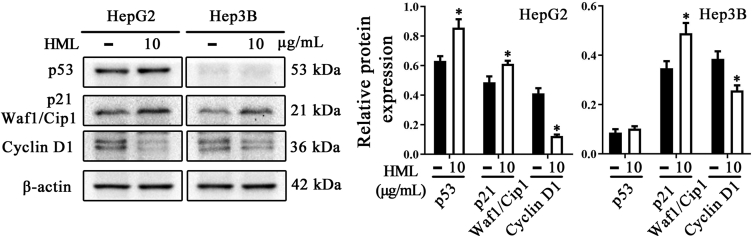
Effects of HML on p53, p21 and Cyclin D1 protein expression. Proliferation-associated proteins were measured by Western blot analysis in HepG2 and Hep3B cells. Statistical analysis was performed using a one–way ANOVA, ∗*P* < 0.05.

**Fig. 3:**
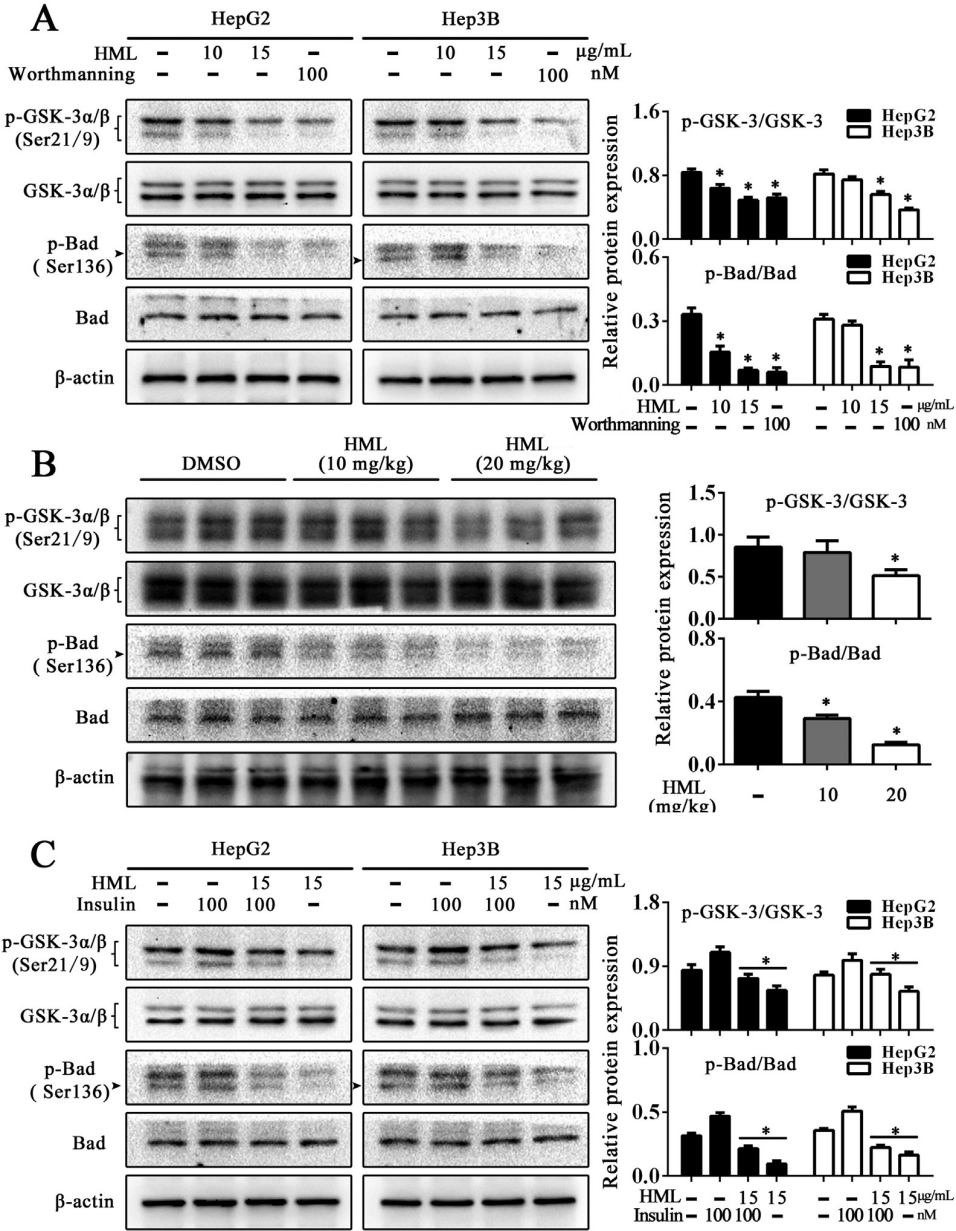
HML inhibits Akt-downstream proteins activity in HCC cells and tumor tissues. Effects of HML on Bad and GSK-3α/β were analyzed by Western blot in HepG2 and Hep3B cells. (A) HML treatment inhibited the phosphorylation of Bad and GSK-3α/β. (B) Similar results were obtained in the tumor tissues of nude mouse models. (C) HepG2 and Hep3B were incubated with DMSO (1‰ V/V), 100 nM insulin or 15 μg/mL HML alone or in combination for 6 h. The inhibition of HML on Bad and GSK-3α/β was partially reversed by insulin stimulation. Statistical analysis was performed using a one-way ANOVA, ∗*P* < 0.05.

**Fig. 4:**
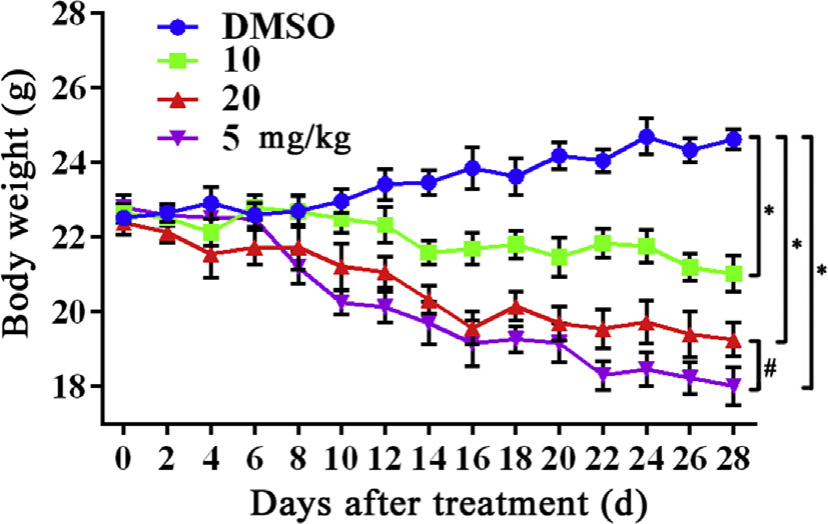
Effect of HML on HepG2-bearing nude mice model body weight. After treatment of HML, nude mice were monitored body weight every two days. HML and CDDP treatment induced weight loss as compared to DMSO-treated controls. Moreover, CDDP treatment showed more intense effect than HML on body weight. Statistical analysis was performed by ANOVA, ∗*P* < 0.05, compared to DMSO-treated group; ^#^*P* < 0.05 compared to CDDP-treated group.

The data in this article describes the anticancer property of α-Humulene (HML) on hepatocellular carcinoma (HCC). Chemical elucidation of HML is reported in [1]. The physiological indexes of HCC-bearing nude mice model were also provided. Fig. 1 describes the selective cytotoxicity of HML in different HCC cell lines or hepatocyte. Fig. 2 describes the effect on p53 function assessed in HML-sensitive cell lines. Fig. 3 describes the specific inhibition of HML on Akt downstream proteins activity (data for Akt inhibition can be found in [1]). Fig. 4 describes the body weight of HCC-bearing nude mice over the period administered by HML or DMSO. Table 1, Table 2 describes the physiological metrics of HCC-bearing and non-tumor nude mice.

**Table 1:** Effect of HML on vital organs in tumor-bearing mice. (mean ± SD, n = 5).

Group	Dose (mg/kg)	Organ index		
		Spleen index	Liver index	Renal index
Non-tumor	–	0.36 ± 0.09	4.65 ± 0.63	1.41 ± 0.22
DMSO	–	3.16 ± 0.25^a^	7.38 ± 0.68^a^	1.42 ± 0.20
HML	10	2.78 ± 0.26^a^	6.42 ± 0.82^a^	1.37 ± 0.24
HML	20	2.62 ± 0.19^a^	6.33 ± 0.70^a^^,^	1.40 ± 0.19
CDDP	5	2.94 ± 0.24^a^	7.69 ± 1.08^a^	1.31 ± 0.38^a^^,^^b^

a *P* < 0.05, compared to non-tumor group.

b *P* < 0.05, compared to DMSO-treated group.

**Table 2:** Effect of HML on blood biochemistry in tumor-bearing mice. (mean ± SD, n = 5).

Group	Dose (mg/kg)	Blood biochemical parameters					
		ALT (U/L)	AST (U/L)	ALP (U/L)	BUN (mmol/L)	CREA (μmol/L)	UA (μmol/L)
Non-tumor	–	68.27 ± 10.05	176.10 ± 34.80	113.42 ± 36.63	5.98 ± 1.87	19.07 ± 2.14	160.43 ± 31.66
DMSO	–	64.85 ± 19.59	180.26 ± 41.33	125.68 ± 29.87	7.49 ± 1.22	20.58 ± 1.58	180.58 ± 48.23
HML	10	72.92 ± 13.85	160.58 ± 38.48	132.64 ± 31.58	6.72 ± 2.06	22.07 ± 1.84	167.91 ± 28.59
HML	20	74.58 ± 22.64	218.11 ± 44.92	122.58 ± 34.59	7.45 ± 1.59	17.86 ± 2.35	203.59 ± 38.04
CDDP	5	103.92 ± 26.25	257.27 ± 37.34^a^	102.32 ± 42.59	16.21 ± 3.56^a^	46.59 ± 10.59^a^	302.57 ± 52.72^a^

a *P* < 0.05, compared to non-tumor group.

## 2 Experimental design, materials and methods

### 2.1 Cell culture

Huh7, SMMC-7721, HepG2 and Hep3B cells are HCC cells lines of human origin. L-02 cells are normal hepatic cell line. HepG2 and Hep3B were purchased from the American Type Culture Collection (Manassas, VA, USA); Huh7, SMMC-7721 and L-02 were purchased from the Library of Typical Culture of Chinese Academy of Sciences (Shanghai, China). After cell thawing, the cells were cultured in Dulbecco's modified Eagle's medium (DMEM; Sigma-Aldrich, Shanghai, China), supplemented with 10% fetal bovine serum and 1% penicillin/streptomycin (Hyclone, UT, USA). All cells were maintained in 25 cm^2^ or 75 cm^2^ cell culture flasks in a humidified atmosphere containing 5% CO_2_ at 37 °C.

### 2.2 Cell cytotoxicity by Cell Counting Kit-8 assay

Cell Counting Kit-8 (CCK-8) assay was performed following the kit protocol (Beyotime, Shanghai, China). Equal numbers of Huh7, SMMC-7721, HepG2, Hep3B and L-02 cells (1 × 10^4^/well) were seeded into 96-well plates and allowed to grow for 24 h, and then the cells were incubated with DMSO (Dimethyl sulfoxide, 1‰ V/V), HML for 12 hours while DMSO-treated group was served as control. Following the treatment time, mediums were removed, and cells were then incubated in fresh DMEM with CCK-8 for additional 3 h before measuring the optical density under 450 nm by spectrophotometer (Thermo Scientific, Vantaa, Finland). Cell inhibition ratio was established utilizing GraphPad Prism 6.0 Software and the IC_50_ value is defined as 50% inhibitory concentrations. As shown in Fig. 1, the IC_50_ values of HML were 16.18 ± 1.84, 19.13 ± 2.03, 11.22 ± 1.25 and 13.78 ± 1.46 μg/mL in Huh7, SMMC-7721, HepG2 and Hep3B respectively and 114.90 ± 3.52 μg/mL in normal hepatocytes (L-02) in 12 h, suggesting cancer-selective cytotoxicity.

### 2.3 Protein preparation and Western blot analysis

HepG2 and Hep3B (5 × 10^5^/dishes) cells were seeded into 100 mm × 20 mm cell culture dishes and allowed to grow to confluency of 60–70%. Then the cells were incubated with designated solutions for 6 or 12 h while DMSO-treated group was served as control. Following the treatment time, cells or xenografts were collected and lysed in RIPA containing phenylmethanesulfonyl fluoride and PhosSTOP (Solarbio, Beijing, China). The supernatant containing protein was collected and stored at −80 °C until use. The measurement of protein contents was performed with a Bicinchoninic acid (BCA) kit (Beyotime, Shanghai, China). Equivalent amounts of the proteins (20 μg total protein from cells for p53 and p21; 25 μg total protein from cells for GSK-3 and Bad; 40 μg total protein from xenografts for GSK-3 and Bad) were separated by SDS-PAGE and transferred to polyvinylidine difluoride membranes (Bio-Rad, CA, USA). The membranes were blocked by 5% skim milk in tris-buffered saline with tween 20 (0.5%) for 2 h. Subsequently, the membranes were incubated with the primary antibody at 4 °C overnight and incubated with the secondary antibody at 37 °C for 2 h. The Horseradish Peroxidase-electrochemiluminescence (HRP ECL) system (Beyotime, Shanghai, China) was used to visualize the protein band and the gray value was analyzed by Quantity One software. As shown in Fig. 2, the HML treatment enhanced the expression of the antiproliferative proteins p53 and p21, with an accompanying decrease in Cyclin D1 expression in HepG2 cells. As expected, p53 was undetectable in Hep3B cells, but the induction of Cyclin D1 expression was decreased accompanying a slight increase of p21. GSK-3β and Bad were typical phosphorylated target of Akt involving in resistance of apoptosis and proliferation in neoplastic processes. Fig. 3A showed that HML treatment downregulated the phosphorylation of GSK-3α/β and Bad, most notably in HepG2 cells. Wortmannin was included as a known PI3K inhibitor [2] in these assays and showed comparable levels of p-GSK-3α/β and p-Bad inhibition in HepG2 cells. We further measured the levels of p-GSK-3α/β and p-Bad in xenograft tissue. Similarly, HML administration inhibited p-Bad and p-GSK-3α/β levels in the tumor tissues of nude mouse models (Fig. 3B). To investigate the specific inhibition of Akt and its downstream proteins mediated by HML, we used insulin to stimulate p-Akt [3] in HepG2 and Hep3B cells co-treated with a range of HML concentrations. Fig. 3C showed that the inhibition of p-Bad and p-GSK-3α/β by HML was partially reverse by insulin treatment, which led to increased p-Bad, and p-GSK-3α/β levels.

### 2.4 HCC-bearing nude mice model and in vivo administration

HepG2-bearing nude mouse model was established as described previously [4]. Briefly, 200 μL of HepG2 cells (1 × 10^7^ per mouse) were subcutaneously transplanted into the right flank of the nude mice (BALB/c, SPF grade, Female, 16–18 g, 4–5 weeks old). When tumors reached 100 mm^3^, the animals were randomized in 4 groups (n = 5 mice per group) and treated intraperitoneal injection (i.p.) with HML (10, 20 mg/kg/2 days) for 4 weeks; Cisplatin (CDDP, 5 mg/kg, i.p.) at day 1, 4, 7, 10, 13 and 16. Body weight was measured every 2 days. At the endpoint of HML therapy, all mice were sacrificed; whole blood samples were collected for hematology; vital organs were dissected out and weighed; transplanted tumors were removed for assessments. All protocols involved in our animal experiments were in accordance with the protocols approved by the Animal Care and Use Committee of South-Central University for Nationalities (Wuhan, China). To detect potential toxic side-effects, body weight was monitored every two days and collected whole blood samples as well as vital organs for hematology and organ index analysis. Although HML and CDDP treatment were not lethal, both compounds increased weight loss in the mice, compared to DMSO-treated controls. The weight loss was more pronounced in CDDP compared to HML treated mice (Fig. 4). No obvious differences in organ indices were evident between DMSO- and HML-treated groups, while CDDP decreased the renal index compared to non-tumor and DMSO-treated groups. Meanwhile, HepG2 bearing remarkably increased spleen and liver index suggested that transplanted tumors could cause splenomegaly and hepatomegaly (Table 1). In hematology analysis, alanine aminotransferase (ALT), aspartate aminotransferase (AST), alkaline phosphatase (ALP), blood urea nitrogen (BUN), creatinine (CREA) and uric acid (UA) were tested. No obvious differences in blood biochemical parameters were evident between DMSO- and HML-treated groups. However, CDDP significantly increased AST, BUN, CREA and UA, indicated that CDDP had a greater damage of liver and kidney function (Table 2).

### 2.5 Statistical analysis

All data are shown as mean ± SD from three independent experiments. GraphPad Prism 6.0 software was used for analysis. Statistically differences were analyzed using one-way analysis of variance (ANOVA) and *P*-values < 0.05 were considered significant.
